# Supplementary material for: Hybrid mass spectrometry approaches in glycoprotein analysis and their usage in scoring biosimilarity
Source: Nat Commun. 2016 Nov 8;7:13397. doi: 10.1038/ncomms13397 (PMC5105167; doi:10.1038/ncomms13397)
Supplement: Supplementary Information — Supplementary Figures 1 - 3, Supplementary Table 1, Supplementary Note 1 and Supplementary References [file ncomms13397-s1.pdf]

Supplementary Information

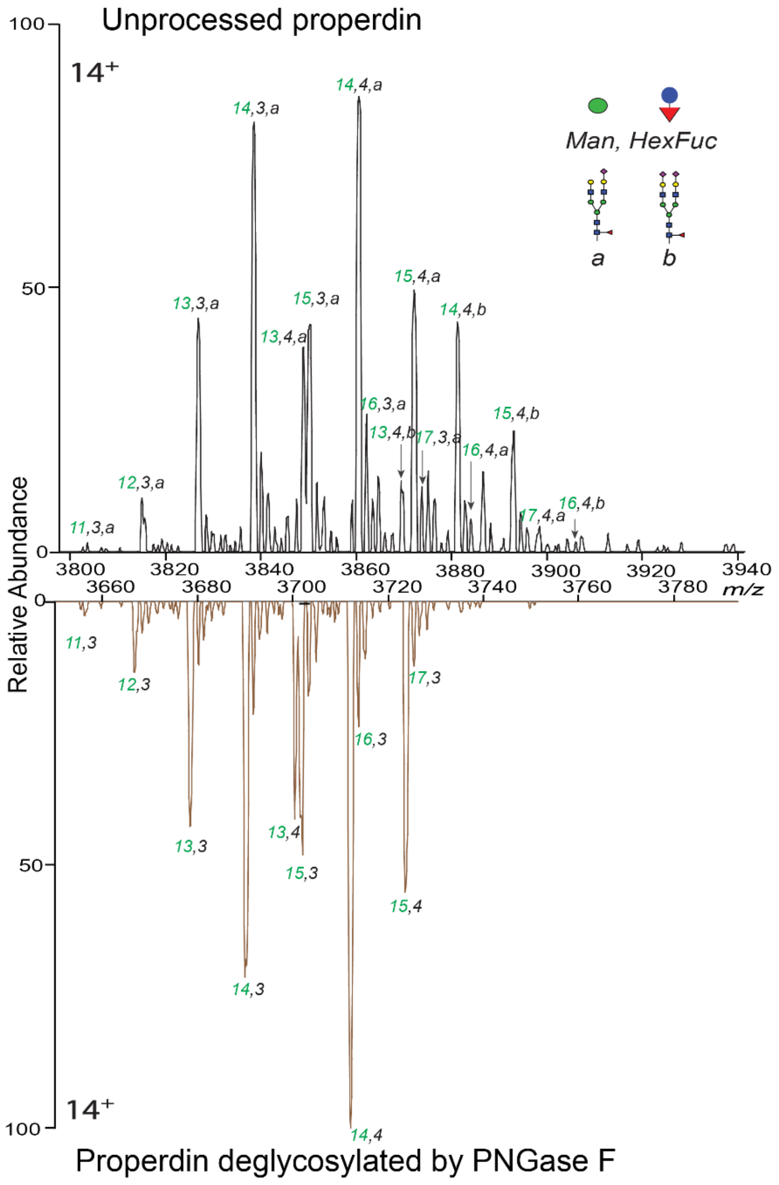

Supplementary Figure 1. Native mass spectra of properdin, before and after PNGaseF treatment, zoomed in on the ions of charge state $[M+14H]^{14+}$. Notably, the proteoform profile of *N*-deglycosylated properdin remained very similar to the untreated wild-type sample. This shows that the main cause of the heterogeneity on properdin is not *N*-glycosylation.

a)

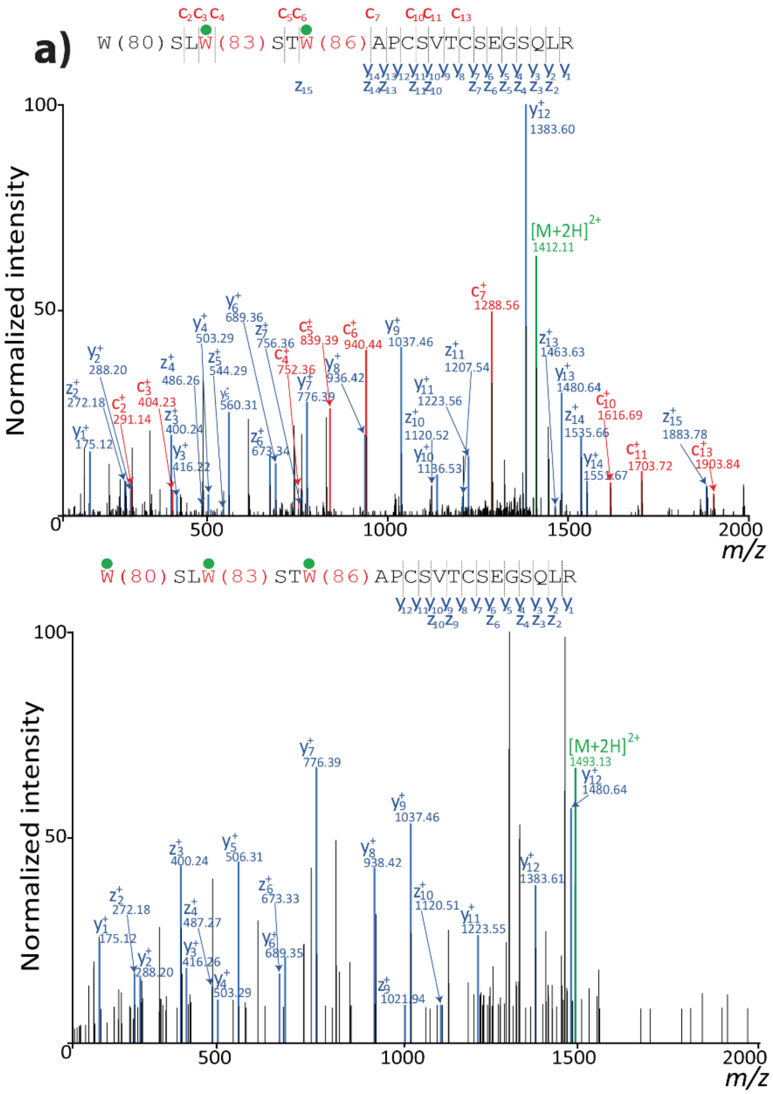
W(80)SLW(83)STW(86)APCSVTCSEGSQLR
Normalized intensity
m/z
[M+2H]2+
1412.11
y12+
1383.60
c7+
1288.56
W(80)SLW(83)STW(86)APCSVTCSEGSQLR
Normalized intensity
m/z
[M+2H]2+
1493.13
y12+
1480.64
y7+
776.39
y9+
1037.46

b)

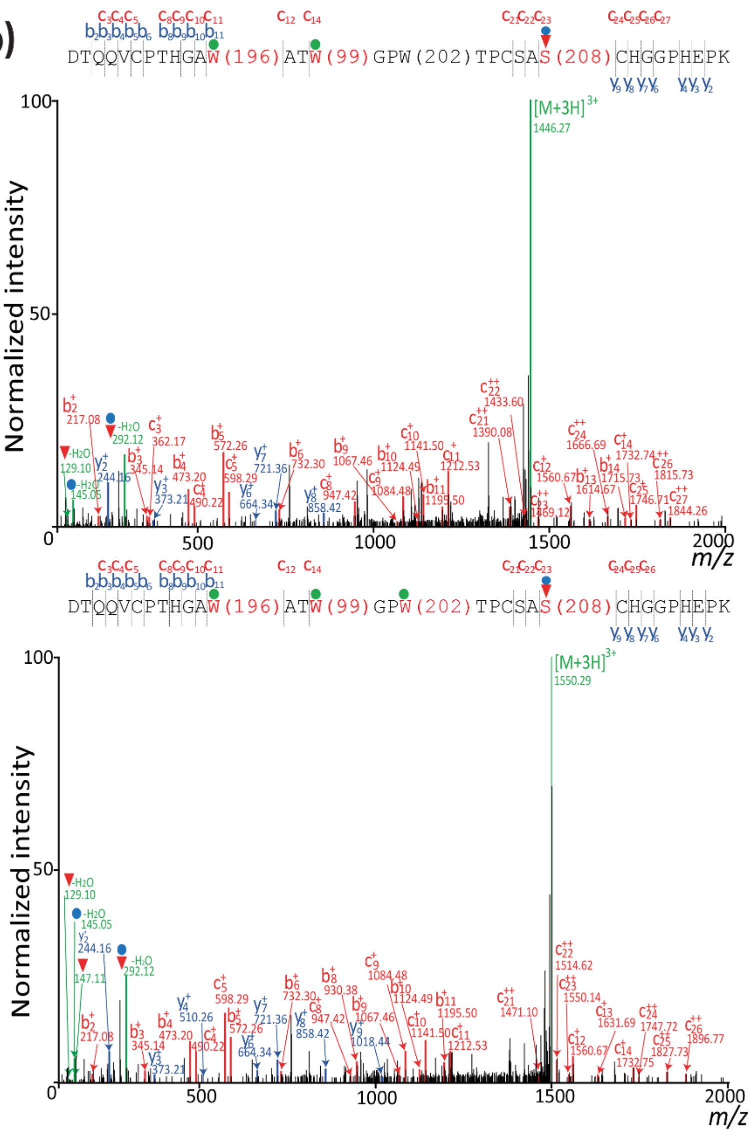
DTQQVCPTHGAW(196)ATW(99)GPW(202)TPCSAS(208)CHGGPHEPK
Normalized intensity
100
50
0
500
1000
1500
2000
m/z
[M+3H]3+
1446.27
DTQQVCPTHGAW(196)ATW(99)GPW(202)TPCSAS(208)CHGGPHEPK
Normalized intensity
100
50
0
500
1000
1500
2000
m/z
[M+3H]3+
1550.29

c)

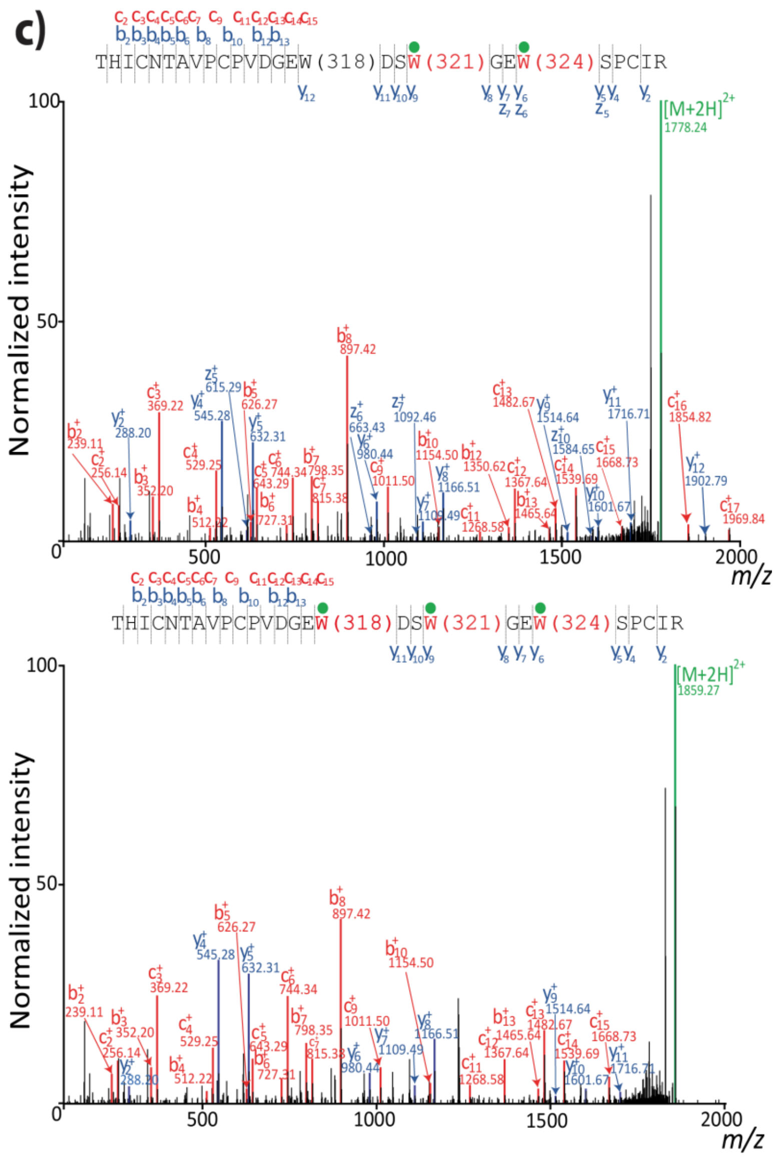

Supplementary Figure 2. EThcD MS2 spectra of peptides derived from a proteolytic digestion of properdin. Six selected MS2 spectra are shown, which are acquired for *C*-mannosylated and *O*-glycosylated peptides with precursor *m/z* of 995.43, 941.41 (a), 722.97, 749.98 (b), 929.64 and 889.13 (c). Fragmentation patterns conclusively confirm the identity of the peptides and the composition of PTMs. Three new discovered *C*-mannosylation sites are located at W80, W202 and W318. Since all of these sites are occupied only partially, we also provide MS2 spectra of non-occupied variants. All spectra are interpreted by using the software Byonic and further validated by manual inspection.

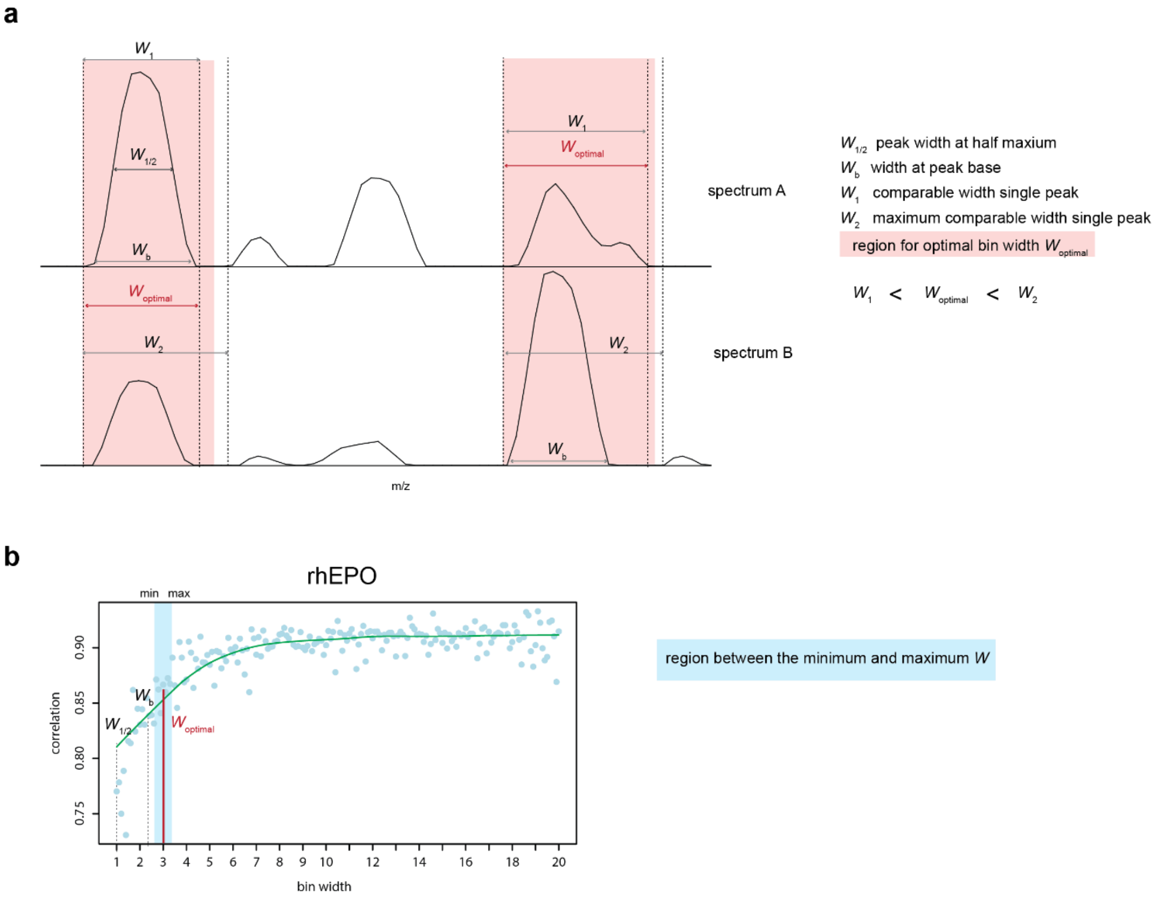

Supplementary Figure 3. (a), Illustration of the ideal bin width ($W_{optimal}$) that needs to be chosen close to W_1, which is the minimum width to include two single peaks to be compared, and no larger than W_2, which is the maximum width to include two single peaks to be compared. (b) Verification of the selected bin width. Plotted are the correlation values over a series of bin widths (1 to 20 *m/z* with a step size of 0.1) with a local polynomial regression fitting line shown in green, the chosen bin width is located close to the region of inflection.

Supplementary Table 1: Quantitative Glycan Profiling of Intact rhEPO by native MS.

No.	mass (Da)	relative abundance (%)	HexNAc	Hex	Fuc	Sia	Acetyl	hydroxyl	glycan (Da)	calculated glycan (Da)	dev
1	26087.73	0.69	17	19	3	3	0	0	7851.74	7847.22	4.52
2	26123.53	0.04	17	21	1	3	0	1	7887.54	7895.22	7.68
3	26308.28	0.13	16	18	3	5	0	0	8072.29	8064.40	7.89
4	26374.33	1.10	17	19	3	4	0	0	8138.34	8138.48	0.14
5	26412.01	0.44	17	21	1	4	0	1	8176.02	8186.48	10.46

6	26448.76	0.26	16	19	3	5	0	0	8212.77	8226.54	13.77
7	26455.77	0.15	16	19	1	6	0	0	8219.78	8225.52	5.74
8	26513.08	0.57	17	18	3	5	0	0	8277.09	8267.60	9.49
9	26533.19	1.21	16	21	3	4	1	0	8297.20	8301.57	4.37
10	26586.85	1.79	16	18	3	6	0	0	8350.86	8355.66	4.80
11	26606.60	0.17	16	19	2	6	0	0	8370.61	8371.66	1.04
12	26636.95	1.86	16	21	2	5	0	0	8400.96	8404.69	3.72
13	26672.76	0.01	17	19	3	5	0	0	8436.77	8429.74	7.03
14	26678.96	0.11	16	21	2	5	1	0	8442.97	8446.69	3.72
15	26752.57	0.03	16	19	3	6	0	0	8516.58	8517.80	1.22
16	26826.92	1.24	16	21	3	5	1	0	8590.93	8592.83	1.90
17	26963.99	2.21	17	19	3	6	0	0	8728.00	8721.00	7.00
18	27116.10	0.39	16	21	3	6	1	0	8880.11	8884.09	3.97
19	27152.78	0.20	19	21	3	4	1	0	8916.79	8911.16	5.63
20	27261.66	0.99	19	21	2	5	0	1	9025.67	9030.27	4.60
21	27316.45	0.11	17	21	3	6	1	0	9080.46	9087.28	6.82
22	27360.58	0.21	18	20	3	6	1	0	9124.59	9128.33	3.75
23	27444.93	0.13	19	21	3	5	1	0	9208.94	9202.41	6.53
24	27493.48	1.52	16	20	3	8	0	0	9257.49	9262.46	4.97
25	27514.72	0.02	18	22	2	6	0	1	9278.73	9280.48	1.75
26	27556.12	1.81	19	22	3	5	0	0	9320.13	9322.56	2.43
27	27582.50	0.00	17	21	3	7	0	0	9346.51	9336.54	9.97
28	27625.99	4.17	16	19	3	9	0	0	9390.00	9391.58	1.58
29	27682.61	0.31	16	21	3	8	0	1	9446.62	9440.60	6.02
30	27701.15	0.45	17	20	3	8	0	0	9465.16	9465.66	0.50
31	27738.07	0.07	19	21	3	6	1	0	9502.08	9493.67	8.41
32	27756.80	0.21	16	18	3	10	0	0	9520.81	9520.69	0.12
33	27817.47	0.02	18	21	3	7	1	0	9581.48	9581.73	0.25
34	27836.13	1.60	17	19	3	9	0	0	9600.14	9594.77	5.37
35	27917.35	5.67	18	20	3	8	0	1	9681.36	9684.85	3.49
36	27963.29	0.61	17	18	3	10	0	0	9727.30	9723.89	3.41
37	27971.65	1.78	17	19	2	10	0	0	9735.66	9739.89	4.22
38	27993.07	1.66	17	20	3	9	0	0	9757.08	9756.91	0.17
39	28016.90	0.52	19	21	3	7	1	0	9780.91	9784.93	4.02
40	28046.66	0.76	19	23	3	6	1	0	9810.67	9817.96	7.28
41	28098.37	0.02	16	20	3	10	0	1	9862.38	9860.98	1.40
42	28122.01	2.18	17	19	3	10	0	0	9886.02	9886.03	0.01
43	28143.34	0.55	17	20	2	10	0	0	9907.35	9902.03	5.33
44	28164.58	1.22	17	21	3	9	0	1	9928.59	9935.06	6.46
45	28209.51	3.90	18	20	3	9	0	1	9973.52	9976.11	2.59
46	28231.77	1.13	18	22	3	8	0	0	9995.78	9993.14	2.64
47	28254.38	3.12	16	21	3	10	0	1	10018.39	10023.12	4.73
48	28283.21	14.21	17	20	3	10	0	0	10047.22	10048.17	0.95

49	28303.51	0.71	17	20	3	10	0	1	10067.52	10064.17	3.34
50	28325.90	3.72	17	20	3	10	1	0	10089.91	10090.17	0.26
51	28337.95	0.99	19	22	2	8	1	0	10101.96	10092.19	9.78
52	28367.58	0.10	17	20	3	10	2	0	10131.59	10132.17	0.58
53	28379.64	0.39	15	21	3	11	1	0	10143.65	10137.18	6.47
54	28410.54	1.36	17	20	3	10	3	0	10174.55	10174.17	0.38
55	28449.96	1.89	17	21	3	10	0	0	10213.97	10210.31	3.66
56	28484.90	1.73	18	20	3	10	0	0	10248.91	10251.37	2.45
57	28517.85	1.71	18	21	2	10	0	1	10281.86	10283.37	1.51
58	28544.85	0.02	15	22	3	11	1	0	10308.86	10299.32	9.54
59	28575.19	34.38	17	20	3	11	0	0	10339.20	10339.43	0.23
60	28595.52	4.20	17	20	3	11	0	1	10359.53	10355.43	4.10
61	28616.91	13.59	17	22	3	10	0	0	10380.92	10372.46	8.46
62	28648.78	7.47	18	21	3	10	0	0	10412.79	10413.51	0.72
63	28660.29	2.44	18	21	3	10	0	1	10424.30	10429.51	5.20
64	28702.61	4.37	18	21	3	10	1	0	10466.62	10455.51	11.11
65	28723.27	0.14	19	22	3	9	0	0	10487.28	10487.59	0.31
66	28742.46	3.55	17	21	3	11	0	0	10506.47	10501.57	4.90
67	28778.41	4.52	18	20	3	11	0	0	10542.42	10542.62	0.20
68	28811.26	1.20	18	22	3	10	0	0	10575.27	10575.65	0.38
69	28823.54	1.08	16	21	3	12	0	0	10587.55	10589.63	2.08
70	28866.20	21.00	17	20	3	12	0	0	10630.21	10630.69	0.48
71	28886.48	2.22	19	23	3	9	0	0	10650.49	10649.73	0.76
72	28908.56	10.11	17	22	3	11	0	0	10672.57	10663.71	8.85
73	28940.54	45.01	18	21	3	11	0	0	10704.55	10704.77	0.21
74	28960.51	2.55	18	21	3	11	0	1	10724.52	10720.77	3.76
75	28982.94	14.98	18	21	3	11	1	0	10746.95	10746.77	0.18
76	29015.01	2.06	19	22	3	10	0	0	10779.02	10778.85	0.18
77	29025.73	5.98	17	21	3	12	0	0	10789.74	10792.83	3.09
78	29067.26	6.33	18	20	3	12	0	0	10831.27	10833.88	2.61
79	29087.73	0.02	18	20	3	12	0	1	10851.74	10849.88	1.86
80	29109.21	3.82	18	20	3	12	1	0	10873.22	10875.88	2.67
81	29137.45	0.31	19	21	3	11	0	0	10901.46	10907.96	6.51
82	29153.91	0.17	17	20	3	13	0	0	10917.92	10921.94	4.03
83	29168.92	0.07	19	22	2	11	0	1	10932.93	10939.96	7.03
84	29189.22	0.67	19	21	3	11	1	0	10953.23	10949.96	3.27
85	29231.91	65.89	18	21	3	12	0	0	10995.92	10996.02	0.11
86	29251.63	8.01	18	21	3	12	0	1	11015.64	11012.02	3.61
87	29273.70	24.35	18	21	3	12	1	0	11037.71	11038.02	0.32
88	29304.90	35.14	19	22	3	11	0	0	11068.91	11070.10	1.19
89	29347.54	7.24	19	22	3	11	1	0	11111.55	11112.10	0.56
90	29357.74	3.00	18	20	3	13	0	0	11121.75	11125.14	3.39
91	29388.22	4.15	18	22	3	12	0	0	11152.23	11158.17	5.94

92	29400.66	3.76	18	21	2	13	0	1	11164.67	11157.14	7.53
93	29432.92	3.79	19	21	3	12	0	0	11196.93	11199.22	2.29
94	29440.02	2.01	19	22	2	12	0	0	11204.03	11215.22	11.19
95	29460.69	0.28	19	22	2	12	0	1	11224.70	11231.22	6.51
96	29474.31	1.45	20	20	3	12	0	0	11238.32	11240.27	1.96
97	29523.32	36.37	18	21	3	13	0	0	11287.33	11287.28	0.05
98	29543.20	4.02	18	21	3	13	0	1	11307.21	11303.28	3.93
99	29565.15	13.89	19	20	3	13	0	0	11329.16	11328.33	0.83
100	29596.87	71.94	19	22	3	12	0	0	11360.88	11361.36	0.49
101	29617.43	4.96	19	22	3	12	0	1	11381.44	11377.36	4.08
102	29639.77	27.40	19	22	3	12	1	0	11403.78	11403.36	0.42
103	29671.07	15.85	20	23	3	11	0	0	11435.08	11435.44	0.36
104	29683.34	5.09	18	22	3	13	0	0	11447.35	11449.42	2.07
105	29724.18	6.19	19	21	3	13	0	0	11488.19	11490.48	2.29
106	29743.08	0.02	19	22	2	13	0	0	11507.09	11506.48	0.62
107	29753.97	0.65	19	23	3	12	0	0	11517.98	11523.50	5.53
108	29764.71	6.36	20	20	3	13	0	0	11528.72	11531.53	2.81
109	29798.01	0.94	20	22	3	12	0	0	11562.02	11564.56	2.54
110	29808.65	2.71	18	21	3	14	0	0	11572.66	11578.54	5.88
111	29847.70	1.65	18	23	3	13	0	0	11611.71	11611.57	0.14
112	29888.02	100.00	19	22	3	13	0	0	11652.03	11652.62	0.59
113	29908.80	10.94	19	22	3	13	0	1	11672.81	11668.62	4.19
114	29930.46	37.81	20	21	3	13	0	0	11694.47	11693.67	0.79
115	29962.26	39.07	20	23	3	12	0	0	11726.27	11726.70	0.43
116	29970.65	6.82	19	22	3	13	2	0	11734.66	11736.62	1.96
117	30004.13	8.71	20	23	3	12	1	0	11768.14	11768.70	0.56
118	30014.60	5.75	19	22	3	13	3	0	11778.61	11778.62	0.01
119	30036.90	13.12	19	22	4	13	0	0	11800.91	11798.76	2.15
120	30054.99	10.48	19	23	3	13	0	0	11819.00	11814.76	4.24
121	30079.85	1.32	19	22	4	13	1	0	11843.86	11840.76	3.09
122	30083.01	1.01	21	22	3	12	2	0	11847.02	11851.75	4.73
123	30097.46	4.22	19	22	4	13	1	1	11861.47	11856.76	4.71
124	30122.45	1.21	19	22	4	13	2	0	11886.46	11882.76	3.70
125	30129.20	0.35	20	22	3	13	1	0	11893.21	11897.81	4.60
126	30139.21	4.43	19	23	3	13	2	0	11903.22	11898.76	4.46
127	30179.70	55.70	19	22	3	14	0	0	11943.71	11943.88	0.17
128	30199.45	5.65	19	23	4	13	0	0	11963.46	11960.90	2.56
129	30221.87	21.28	19	22	3	14	1	0	11985.88	11985.88	0.01
130	30254.31	60.48	20	23	3	13	0	0	12018.32	12017.96	0.36
131	30271.50	3.64	20	22	4	13	1	0	12035.51	12043.96	8.45
132	30296.61	22.81	20	23	3	13	1	0	12060.62	12059.96	0.66
133	30327.08	13.41	19	22	4	14	0	0	12091.09	12090.02	1.07
134	30346.60	6.18	20	20	3	15	0	0	12110.61	12114.05	3.43

135	30370.65	7.68	20	22	3	14	0	0	12134.66	12147.07	12.41
136	30376.63	1.80	20	22	3	14	0	0	12140.64	12147.07	6.43
137	30401.98	4.76	20	22	3	14	0	1	12165.99	12163.07	2.92
138	30421.98	4.54	20	22	3	14	1	0	12185.99	12189.07	3.08
139	30453.91	1.42	21	23	3	13	0	0	12217.92	12221.15	3.23
140	30463.59	0.57	20	22	3	14	2	0	12227.60	12231.07	3.47
141	30470.76	1.97	19	22	5	14	0	0	12234.77	12236.16	1.40
142	30495.72	1.31	21	23	3	13	1	0	12259.73	12263.15	3.42
143	30510.54	0.96	19	22	3	15	1	0	12274.55	12277.14	2.59
144	30544.66	40.32	20	23	3	14	0	0	12308.67	12309.21	0.54
145	30564.12	3.53	20	23	3	14	0	1	12328.13	12325.21	2.92
146	30587.23	13.58	21	22	3	14	0	0	12351.24	12350.27	0.98
147	30619.77	48.16	21	24	3	13	0	0	12383.78	12383.29	0.48
148	30638.60	1.95	20	20	3	16	0	0	12402.61	12405.30	2.70
149	30662.10	16.44	22	23	3	13	0	0	12426.11	12424.35	1.76
150	30693.82	20.67	20	22	3	15	0	1	12457.83	12454.33	3.50
151	30702.09	2.08	20	24	3	14	0	0	12466.10	12471.36	5.25
152	30712.83	3.06	20	22	3	15	1	0	12476.84	12480.33	3.49
153	30735.38	3.33	21	23	3	14	0	0	12499.39	12512.41	13.02
154	30746.00	1.55	21	23	3	14	0	0	12510.01	12512.41	2.40
155	30768.18	1.98	21	23	3	14	0	1	12532.19	12528.41	3.78
156	30787.56	4.10	21	25	3	13	0	0	12551.57	12545.44	6.13
157	30824.78	0.89	22	22	3	14	1	0	12588.79	12595.46	6.67
158	30837.29	1.29	20	23	3	15	0	0	12601.30	12600.47	0.83
159	30859.68	0.47	23	23	3	13	0	0	12623.69	12627.54	3.86
160	30872.49	1.05	21	22	3	15	0	0	12636.50	12641.53	5.03
161	30910.06	30.75	21	24	3	14	0	0	12674.07	12674.55	0.48
162	30916.16	0.55	21	22	3	15	1	0	12680.17	12683.53	3.36
163	30929.27	1.30	19	22	4	16	0	1	12693.28	12688.54	4.74
164	30952.66	12.06	22	23	3	14	0	0	12716.67	12715.60	1.06
165	30985.05	34.95	20	22	3	16	0	1	12749.06	12745.59	3.48
166	31027.04	13.13	23	22	3	14	1	0	12791.05	12798.66	7.61
167	31059.21	8.64	24	23	3	13	0	0	12823.22	12830.74	7.52
168	31064.38	2.94	19	22	3	17	0	1	12828.39	12833.65	5.26
169	31078.62	2.57	21	23	3	15	1	0	12842.63	12845.67	3.04
170	31111.15	2.32	22	24	3	14	0	0	12875.16	12877.75	2.59
171	31132.56	1.28	22	25	2	14	0	0	12896.57	12893.75	2.82
172	31151.74	1.59	23	23	3	14	0	0	12915.75	12918.80	3.05
173	31193.43	0.99	23	23	3	14	1	0	12957.44	12960.80	3.36
174	31224.12	0.15	24	24	3	13	0	0	12988.13	12992.88	4.75
175	31243.03	0.72	22	23	3	15	0	0	13007.04	13006.86	0.17
176	31275.76	23.01	22	25	3	14	0	0	13039.77	13039.89	0.11
177	31295.26	0.44	23	23	4	14	0	0	13059.27	13064.94	5.67

178	31317.63	7.18	23	24	3	14	0	0	13081.64	13080.94	0.70
179	31328.72	0.01	23	25	2	14	0	0	13092.73	13096.94	4.21
180	31350.16	22.41	24	23	3	14	0	0	13114.17	13121.99	7.83
181	31365.46	0.03	21	23	3	16	1	0	13129.47	13136.93	7.46
182	31392.59	6.53	24	25	3	13	0	0	13156.60	13155.02	1.58
183	31425.60	8.67	22	25	4	14	0	0	13189.61	13186.03	3.57
184	31432.73	0.97	20	23	3	17	0	1	13196.74	13198.99	2.25
185	31444.56	1.39	23	23	3	15	0	0	13208.57	13210.06	1.49
186	31474.08	2.24	23	25	3	14	0	0	13238.09	13243.08	4.99
187	31512.09	1.62	24	24	3	14	0	0	13276.10	13284.14	8.04
188	31522.03	1.40	24	24	3	14	0	0	13286.04	13284.14	1.90
189	31567.57	2.79	22	25	3	15	0	0	13331.58	13331.15	0.43
190	31604.78	0.36	25	23	3	14	1	0	13368.79	13367.19	1.60
191	31612.68	0.43	23	22	3	16	1	0	13376.69	13381.17	4.48
192	31641.13	12.73	24	23	3	15	0	0	13405.14	13413.25	8.11
193	31658.99	1.01	24	24	4	14	0	0	13423.00	13430.28	7.28
194	31683.06	3.97	24	25	3	14	0	0	13447.07	13446.28	0.79
195	31715.12	11.74	25	24	3	14	0	0	13479.13	13487.33	8.20
196	31769.95	0.77	23	25	3	15	0	0	13533.96	13534.34	0.39
197	31793.92	2.32	19	22	4	19	0	1	13557.93	13562.31	4.38
198	31832.97	1.26	24	25	4	14	0	0	13596.98	13592.42	4.56
199	31843.28	0.97	24	26	3	14	0	0	13607.29	13608.42	1.13
200	31882.13	1.45	25	25	3	14	0	0	13646.14	13649.47	3.34
201	31889.93	0.37	25	26	2	14	0	0	13653.94	13665.47	11.53
202	31913.96	0.50	23	22	3	17	1	0	13677.97	13672.43	5.54
203	31932.17	2.28	24	23	3	16	0	0	13696.18	13704.51	8.34
204	31978.25	1.07	24	25	3	15	0	0	13742.26	13737.54	4.72
205	32006.08	6.88	24	27	3	14	0	0	13770.09	13770.56	0.48
206	32025.90	1.77	25	25	4	14	0	0	13789.91	13795.62	5.71
207	32050.21	2.36	25	26	3	14	0	0	13814.22	13811.62	2.60
208	32077.80	4.21	26	25	3	14	0	0	13841.81	13852.67	10.86
209	32100.95	0.00	24	24	3	16	0	0	13864.96	13866.65	1.69
210	32121.25	1.14	24	26	3	15	0	0	13885.26	13899.68	14.42
211	32155.21	5.67	24	27	4	14	0	0	13919.22	13916.71	2.51
212	32161.60	0.84	25	25	3	15	0	0	13925.61	13940.73	15.13
213	32177.44	0.62	25	25	3	15	0	0	13941.45	13940.73	0.72
214	32202.94	2.24	25	26	4	14	0	0	13966.95	13957.76	9.19
215	32237.46	0.86	23	24	3	17	1	0	14001.47	13996.72	4.75
216	32249.58	0.63	26	26	3	14	0	0	14013.59	14014.81	1.22
217	32295.73	0.99	24	27	3	15	0	0	14059.74	14061.82	2.08
218	32342.09	1.19	25	26	3	15	0	0	14106.10	14102.87	3.22
219	32371.96	1.30	26	25	3	15	0	0	14135.97	14143.93	7.96
220	32389.71	1.23	26	26	4	14	0	0	14153.72	14160.96	7.24

221	32408.57	0.84	26	27	3	14	0	0	14172.58	14176.95	4.37
222	32445.33	2.05	27	26	3	14	0	0	14209.34	14218.01	8.66
223	32478.36	5.05	25	23	3	17	1	0	14242.37	14240.96	1.41
224	32487.21	0.01	25	26	4	15	0	0	14251.22	14249.02	2.20
225	32500.62	0.88	25	27	3	15	0	0	14264.63	14265.02	0.39
226	32524.99	2.25	24	23	3	18	0	0	14289.00	14287.03	1.97
227	32558.87	5.23	26	27	4	14	0	0	14322.88	14323.10	0.22
228	32606.88	1.23	27	27	3	14	0	0	14370.89	14380.15	9.26
229	32654.10	0.53	28	26	3	14	0	0	14418.11	14421.20	3.09
230	32701.41	0.53	26	27	3	15	0	0	14465.42	14468.21	2.79
231	32735.13	0.47	25	23	3	18	0	0	14499.14	14490.22	8.92
232	32745.19	0.38	26	27	3	15	1	0	14509.20	14510.21	1.01
233	32780.05	0.13	27	26	3	15	1	0	14544.06	14551.26	7.21
234	32805.93	1.53	28	26	4	14	0	0	14569.94	14567.35	2.59
235	32809.73	0.40	28	27	3	14	0	0	14573.74	14583.34	9.60
236	32853.87	0.02	29	26	3	14	0	0	14617.88	14624.40	6.52

Supplementary Note 1: Heterogeneity of properdin

Properdin belongs to the protein superfamily which contains a functional protein module thrombospondin repeat (TSR). These proteins seem to be a major group of acceptor substrates for *C*-mannosylation, however the function of this unusual modification remains unclear. Interestingly, properdin TSR1, 2, 3 and 4 has been found also *O*-fucosylated in the C-X-X(S/T)C-X motif adjacent to the *C*-mannosylation sites (1). *O*-fucosylation plays some role in the folding and secretion of TSR superfamily proteins (e.g., ADAMTS-13 and ADAMTS-like 1) (2), but the functional relationship between *O*-fucosylation and *C*-mannosylation in the same TSR is unknown. For that reason, our specified information about structural details of properdin could be helpful to understand more to this peculiar structures as TSRs are. Figure 6c shows all identified peptide proteoforms of properdin together with their estimated relative abundances. Five of six TSRs presented in the monomer of properdin contain a complex recognition motif for *C*-mannosylation W-X-X-W-X-X-W, only TSR4 contains a sequence motif W-X-X-W-X-X-X, without W or other aromatic residue at position 3+ relative to the second W. Additionally to *C*-mannosylation and *O*-glycosylation sites, there is also one *N*-glycosylation site at TSR6.

More detail look on the graph (c) in Figure 6 shows a certain variability of *C*-mannosylation occupancy in different TSRs. In all TSR structures of properdin the second W in the complex recognition motif W-X-X-W-X-X-W is found always *C*-mannosylated while the first and the third are modified partially with different level of occupancy. The highest level of heterogeneity

can be observed in TSR1. The first W (W80) is modified in less than 0.5% cases. On the other hand, the third W (W86) is occupied for more than 93%. Lower occupancy of the first W (W318) can be also observed in the TSR5 (approx. 9%) while the third W (W324) is fully modified. TSR2 and TSR6 show a different *C*-mannosylation pattern. In most of the cases all three W sites are occupied by mannose, 80% in TSR2 and 97% in TSR6. In contrast with TSR1 and TSR5, the first W in TSR2 and TSR6 (W139 and W382) are modified 100%. The third W in both of these TSRs is not modified in 20% (W145) and 3% (W366) respectively. Similarly to TSR2 and TSR6, TSR3 also contains mannose on the first W (W196) in 100%. However, the third W (W202) is in majority non-modified (69%). The last properdin structure module TSR4 contains two fully occupied *C*-mannosylation sites, W260 and W263. The third W in the sequence motif W-X-X-W-X-X-W is untypically replaced by an amino acid valine. Regarding *O*-glycosylation site-occupancy, TSR1 shows partial *O*-glycosylation (Glc-Fuc) on T92 (55%). *O*-glycosylation sites T151, S208 and T272 in TSR2, 3 and 4 are exclusively modified by Glc-Fuc. The full picture of proteoform heterogeneity is completed by the information about the existence of *N*-glycosylation on *C*-termini of properdin. N428 is modified by two types of glycan, both of which is a complex biantennary *N*-glycan with a fucosylated core and sialylated antennas.

Supplementary References

1) Gonzalez de Peredo, A., Klein, D., Macek, B., Hess, D., Peter-Katalinic, J. & Hofsteenge, J. C-mannosylation and O-fucosylation of thrombospondin type 1 repeats *Mol. Cell Proteomics* **1,** 11–18 (2002).

2) Wang, L. W., Dlugosz, M., Somerville, R. P. T., Read, M., Haltiwanger, R. S. & Apte, S. S. O-fucosylation of thrombospondin type 1 repeats in ADAMTS-like-1/Punctin-1 regulates secretion: implications for the ADAMTS superfamily *J. Biol. Chem*. **282**, 17024-17031 (2007).
